# Supplementary figures and images for: Charcot-Marie-Tooth type 4B2 demyelinating neuropathy in miniature Schnauzer dogs caused by a novel splicing SBF2 (MTMR13) genetic variant: a new spontaneous clinical model
Source: PeerJ. 2019 Nov 21;7:e7983. doi: 10.7717/peerj.7983 (PMC6875392; doi:10.7717/peerj.7983)

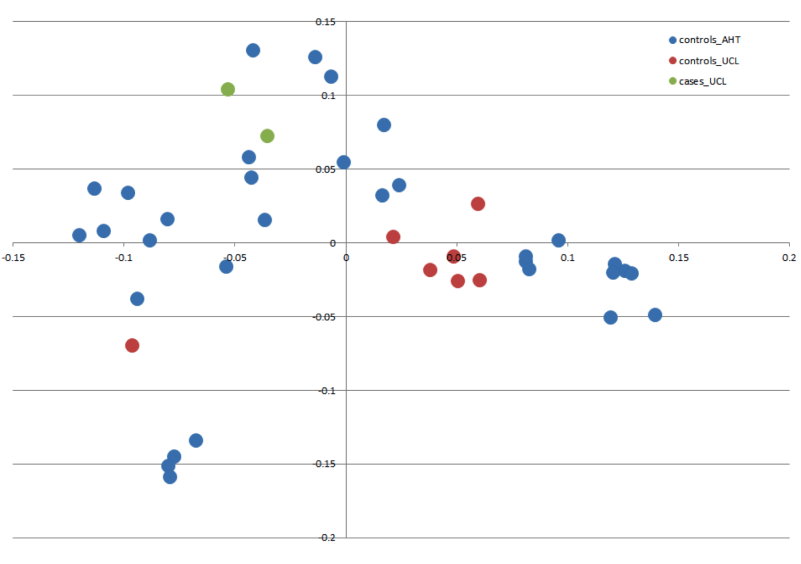

Supplement: File S1 [file peerj-07-7983-s001.png]
