# Supplementary material for: Charcot-Marie-Tooth type 4B2 demyelinating neuropathy in miniature Schnauzer dogs caused by a novel splicing SBF2 (MTMR13) genetic variant: a new spontaneous clinical model
Source: PeerJ. 2019 Nov 21;7:e7983. doi: 10.7717/peerj.7983 (PMC6875392; doi:10.7717/peerj.7983)
Supplement: File S3 [file peerj-07-7983-s003.docx]

*SBF2* gene, chromosome 21, exon 19, *Canis Lupus Familiaris*

Previously known sequence:

TCA GCA CCA GGT GAC TGG GAG AGT GGA AGC AAC AGC

New sequence corresponding to natural abnormal variant:

TCA GCA CCA GTA TTG CAG GAA GTG TAG
