# Supplementary material for: Charcot-Marie-Tooth type 4B2 demyelinating neuropathy in miniature Schnauzer dogs caused by a novel splicing SBF2 (MTMR13) genetic variant: a new spontaneous clinical model
Source: PeerJ. 2019 Nov 21;7:e7983. doi: 10.7717/peerj.7983 (PMC6875392; doi:10.7717/peerj.7983)
Supplement: File S4 [file peerj-07-7983-s004.docx]

**Supplementary file 4**

Genotypes for the *SBF2* c.2363+1 G>T mutation in whole genome sequences of 802 dogs of 162 breeds.

| Breed | Total | w/t | het | hom |
| --- | --- | --- | --- | --- |
| Affenpinscher | 2 | 2 | 0 | 0 |
| Airedale Terrier | 5 | 5 | 0 | 0 |
| Alaskan Husky | 2 | 2 | 0 | 0 |
| Alaskan Malamute | 4 | 4 | 0 | 0 |
| Alpine Dachsbracke | 2 | 2 | 0 | 0 |
| American Bulldog | 2 | 2 | 0 | 0 |
| American Cocker Spaniel | 1 | 1 | 0 | 0 |
| American Staffordshire Terrier | 2 | 2 | 0 | 0 |
| Australian Cattle Dog | 5 | 5 | 0 | 0 |
| Australian Shepherd | 3 | 3 | 0 | 0 |
| Australian Terrier | 1 | 1 | 0 | 0 |
| Basenji | 7 | 7 | 0 | 0 |
| Grand Basset Griffon Vendeen | 1 | 1 | 0 | 0 |
| Basset Hound | 9 | 9 | 0 | 0 |
| Bavarian Hound | 1 | 1 | 0 | 0 |
| Beagle | 8 | 8 | 0 | 0 |
| Bearded Collie | 13 | 13 | 0 | 0 |
| Bedlington Terrier | 1 | 1 | 0 | 0 |
| Berger Blanc Suisse | 1 | 1 | 0 | 0 |
| Berger Picard | 2 | 2 | 0 | 0 |
| Bichon Frise | 6 | 6 | 0 | 0 |
| Black Russian Terrier | 1 | 1 | 0 | 0 |
| Bloodhound | 1 | 1 | 0 | 0 |
| Border Collie | 44 | 44 | 0 | 0 |
| Border Collie Cross | 1 | 1 | 0 | 0 |
| Border Terrier | 9 | 9 | 0 | 0 |
| Boston Terrier | 1 | 1 | 0 | 0 |
| Boxer | 2 | 2 | 0 | 0 |
| Briard | 2 | 2 | 0 | 0 |
| Brussels Griffon | 3 | 3 | 0 | 0 |
| Bull Terrier | 3 | 3 | 0 | 0 |
| Bulldog | 3 | 3 | 0 | 0 |
| Bullmastiff | 2 | 2 | 0 | 0 |
| Cairn Terrier | 4 | 4 | 0 | 0 |
| Cane Corso | 5 | 5 | 0 | 0 |
| Cardigan Welsh Corgi | 1 | 1 | 0 | 0 |
| Cavalier King Charles Spaniel | 6 | 6 | 0 | 0 |
| Central Asian Shepherd dog | 1 | 1 | 0 | 0 |
| Cesky Terrier | 1 | 1 | 0 | 0 |
| Chesapeake Bay Retriever | 1 | 1 | 0 | 0 |
| Chihuahua | 6 | 6 | 0 | 0 |
| Chinese Crested Dog | 3 | 3 | 0 | 0 |
| Chinese Indigenous Dog | 28 | 28 | 0 | 0 |
| Chow Chow | 2 | 2 | 0 | 0 |
| Cocker Spaniel | 1 | 1 | 0 | 0 |
| Collie | 1 | 1 | 0 | 0 |
| Cross Breed | 4 | 4 | 0 | 0 |
| Curly Coated Retriever | 5 | 5 | 0 | 0 |
| Dachshund | 6 | 6 | 0 | 0 |
| Dalmatian | 4 | 4 | 0 | 0 |
| Dandie Dinmont Terrier | 5 | 5 | 0 | 0 |
| Doberman Pinscher | 5 | 5 | 0 | 0 |
| Dogue de Bordeaux | 6 | 6 | 0 | 0 |
| Dutch Shepherd | 1 | 1 | 0 | 0 |
| Elo | 1 | 1 | 0 | 0 |
| English Bulldog | 1 | 1 | 0 | 0 |
| English Cocker Spaniel | 3 | 3 | 0 | 0 |
| English Mastiff | 2 | 2 | 0 | 0 |
| English Setter | 1 | 1 | 0 | 0 |
| English Springer Spaniel | 3 | 3 | 0 | 0 |
| Entlebucher Sennenhund | 8 | 8 | 0 | 0 |
| Eurasier | 2 | 2 | 0 | 0 |
| Field Spaniel | 1 | 1 | 0 | 0 |
| Finnish Lapphund | 2 | 2 | 0 | 0 |
| Flat Coated Retriever | 4 | 4 | 0 | 0 |
| French Bulldog | 7 | 7 | 0 | 0 |
| Friesian Stabyhoun | 2 | 2 | 0 | 0 |
| German Shepherd Dog | 20 | 20 | 0 | 0 |
| German Wirehaired | 1 | 1 | 0 | 0 |
| Giant Schnauzer | 5 | 5 | 0 | 0 |
| Glen of Imaal Terrier | 1 | 1 | 0 | 0 |
| Golden Retriever | 13 | 13 | 0 | 0 |
| Gordon Setter | 1 | 1 | 0 | 0 |
| Great Dane | 2 | 2 | 0 | 0 |
| Great Pyrenees | 1 | 1 | 0 | 0 |
| Greater Swiss Mountain Dog | 6 | 6 | 0 | 0 |
| Greyhound | 7 | 7 | 0 | 0 |
| Griffon Bruxellois | 1 | 1 | 0 | 0 |
| Havanese | 3 | 3 | 0 | 0 |
| Heideterrier | 1 | 1 | 0 | 0 |
| Hovawart | 3 | 3 | 0 | 0 |
| Irish Red and White Setter | 4 | 4 | 0 | 0 |
| Irish Setter | 2 | 2 | 0 | 0 |
| Irish Soft Coated Wheaten Terrier | 1 | 1 | 0 | 0 |
| Irish Terrier | 3 | 3 | 0 | 0 |
| Irish Water Spaniel | 1 | 1 | 0 | 0 |
| Irish Wolfhound | 8 | 8 | 0 | 0 |
| Italian Greyhound | 1 | 1 | 0 | 0 |
| Italian Spinone | 2 | 2 | 0 | 0 |
| Jack Russell Terrier | 8 | 8 | 0 | 0 |
| Jagdterrier | 2 | 2 | 0 | 0 |
| Japanese Akita | 1 | 1 | 0 | 0 |
| Japanese Chin | 1 | 1 | 0 | 0 |
| Karelian Bear dog | 1 | 1 | 0 | 0 |
| Keeshond | 3 | 3 | 0 | 0 |
| Kerry Blue Terrier | 2 | 2 | 0 | 0 |
| Kromfohrlander | 1 | 1 | 0 | 0 |
| Kunming Dog | 10 | 10 | 0 | 0 |
| Labrador Retriever | 14 | 14 | 0 | 0 |
| Lagotto Romagnolo | 10 | 10 | 0 | 0 |
| Lakeland Terrier | 1 | 1 | 0 | 0 |
| Lancashire Heeler | 2 | 2 | 0 | 0 |
| Landseer | 2 | 2 | 0 | 0 |
| Large Munsterlander | 1 | 1 | 0 | 0 |
| Leonberger | 55 | 55 | 0 | 0 |
| Lhasa Apso | 1 | 1 | 0 | 0 |
| Malinois | 7 | 7 | 0 | 0 |
| Maltese | 1 | 1 | 0 | 0 |
| Miniature Bull terrier | 2 | 2 | 0 | 0 |
| Miniature Long Haired Dachshund | 2 | 2 | 0 | 0 |
| Miniature Poodle | 2 | 2 | 0 | 0 |
| Miniature Schnauzer | 25 | 22 | 2 | 1 |
| Miniature Wire Haired Dachshund | 1 | 1 | 0 | 0 |
| Mixed Breed | 8 | 8 | 0 | 0 |
| Newfoundland | 3 | 3 | 0 | 0 |
| Northern Inuit | 2 | 2 | 0 | 0 |
| Norwegian Buhund | 3 | 3 | 0 | 0 |
| Norwich Terrier | 5 | 5 | 0 | 0 |
| Nova Scotia Duck Tolling Retriever | 2 | 2 | 0 | 0 |
| Old English Sheepdog | 2 | 2 | 0 | 0 |
| Otterhound | 2 | 2 | 0 | 0 |
| Papillon | 4 | 4 | 0 | 0 |
| Pembroke Welsh Corgi | 4 | 4 | 0 | 0 |
| Perro de Agua Espanol | 1 | 1 | 0 | 0 |
| Petit Basset Griffon Vendeen | 5 | 5 | 0 | 0 |
| Pomeranian | 4 | 4 | 0 | 0 |
| Poodle | 19 | 19 | 0 | 0 |
| Portuguese Podengo | 1 | 1 | 0 | 0 |
| Portuguese Water Dog | 3 | 3 | 0 | 0 |
| Pug | 22 | 22 | 0 | 0 |
| Rhodesian Ridgeback | 4 | 4 | 0 | 0 |
| Rottweiler | 4 | 4 | 0 | 0 |
| Rough Collie | 1 | 1 | 0 | 0 |
| Saluki | 2 | 2 | 0 | 0 |
| Scottish Deerhound | 3 | 3 | 0 | 0 |
| Scottish Terrier | 6 | 6 | 0 | 0 |
| Shar Pei | 2 | 2 | 0 | 0 |
| Shetland Sheepdog | 4 | 4 | 0 | 0 |
| Shih Tzu | 5 | 5 | 0 | 0 |
| Siberian Husky | 5 | 5 | 0 | 0 |
| Skye Terrier | 2 | 2 | 0 | 0 |
| Sloughi | 3 | 3 | 0 | 0 |
| Smooth Collie | 1 | 1 | 0 | 0 |
| Soft Coated Wheaten Terrier | 2 | 2 | 0 | 0 |
| Spitz (Grossspitz) | 1 | 1 | 0 | 0 |
| St. Bernard | 2 | 2 | 0 | 0 |
| Staffordshire Bull Terrier | 1 | 1 | 0 | 0 |
| Standard Poodle | 2 | 2 | 0 | 0 |
| Standard Schnauzer | 1 | 1 | 0 | 0 |
| Swedish Vallhund | 3 | 3 | 0 | 0 |
| Tibetan Mastiff | 10 | 10 | 0 | 0 |
| Tibetan Spaniel | 1 | 1 | 0 | 0 |
| Tibetan Terrier | 4 | 4 | 0 | 0 |
| Vizsla (smooth coat) | 4 | 4 | 0 | 0 |
| Vizsla (wire-haired) | 1 | 1 | 0 | 0 |
| Weimaraner | 3 | 3 | 0 | 0 |
| Welsh Springer Spaniel | 9 | 9 | 0 | 0 |
| West Highland White Terrier | 20 | 20 | 0 | 0 |
| Whippet | 3 | 3 | 0 | 0 |
| White Shepherd | 1 | 1 | 0 | 0 |
| Wolf | 8 | 8 | 0 | 0 |
| Yorkshire Terrier | 69 | 69 | 0 | 0 |
| Total | 802 | 799 | 2 | 1 |
